# Crosstalk between innate immunity and rumen-fecal microbiota under the cold stress in goats

**DOI:** 10.3389/fimmu.2024.1363664

**Published:** 2024-02-26

**Authors:** Tao Luo, Jiangjiang Zhu, Kerui Li, Yongtao Li, Jun Li, Yu Chen, Hengbo Shi

**Affiliations:** ^1^Institute of Dairy Science, College of Animal Sciences, Zhejiang University, Hangzhou, China; ^2^Qinghai-Tibetan Plateau Animal Genetic Resource Reservation and Utilization Key Laboratory of Sichuan Province, Chengdu, China; ^3^College of Animal Science and Technology, Henan University of Animal Husbandry and Economy, Zhengzhou, China; ^4^Institute of Nanjiang Yellow Goat Sciences, Bazhong, Sichuan, China; ^5^Key Laboratory of Molecular Animal Nutrition, Ministry of Education, Zhejiang University, Hangzhou, China

**Keywords:** immunity, probiotics, cold stress, microbiota, rumen, feces

## Abstract

The balance of the microbiome, which is sensitive to temperature changes, plays a crucial role in maintaining overall health and reducing the risk of diseases. However, the specific mechanisms by which immunity and microbiota interact to adapt to cold stress have yet to be addressed. In this study, Nanjiang Yellow goats were chosen as a model and sampled during the cold (winter, cold stress) and warm (spring) seasons, respectively. Analyses of serum immune factors, as well as the composition of rumen and fecal microbial communities, were conducted to explore the crosstalk between microbiota and innate immunity under cold stress. Significantly increased levels of IgA (*P* < 0.01) were observed in the cold season compared to the warm season. Conversely, the levels of IL-2 (*P* = 0.02) and IL-6 (*P* < 0.01) diminished under cold stress. However, no significant differences were observed in IgG (*P* = 0.89), IgM (*P* = 0.42), and IL-4 (*P* = 0.56). While there were no significant changes in the diversity of bacterial communities between the warm and cold seasons, positive correlations between serum IgA, IL-2, IL-6 concentrations and several genera were observed. Furthermore, the weighted gene co-expression network analysis indicated that the microbiota enriched in the MEbrown module positively correlated with IgA, while the microbiota enriched in the MEblue module positively correlated with IL-2 and IL-6. The strong correlation between certain probiotics, including *Alistipes*, *Bacteroides*, *Blautia*, and *Prevotellaceae*_UCG.004, and the concentration of IL-2, and IL-6 suggests their potential role in immunomodulatory properties. This study provides valuable insights into the crosstalk between microbial communities and immune responses under the challenge of cold stress. Further studies on the immunomodulatory properties of these probiotics would contribute to the development of strategies to enhance the stress resistance of animals for improved overall health and survival.

## Introduction

Emerging evidence suggests that cold stress induces inflammation, adversely affecting animal growth and development (1, 2). For instance, cold exposure impacts mammary blood flow and lactose synthesis in dairy livestock (3, 4), thereby reducing milk secretion and diminishing birth weight and survival of young animals (5). In rodents, cold stress reduces the diversity of the gut microbiota and increases the abundance of potentially pathogenic bacteria (6), suggesting a crosstalk between immunity and microbiota under cold stress. However, the mechanisms by which immunity and microbiota interact to adapt to cold exposure remain unaddressed.

There has been a growing interest in unraveling the intricate relationship between innate immunity and the profile of microbiota (7–9). These microbiota serve multifaceted functions, including defending against pathogens (10), facilitating exogenous metabolism (11), and supporting the maturation of the immune system (12), vital for their sustained survival (13). In the ruminants, maintaining a healthy-balanced microbial community is particularly vital for the reconstruction of rumen and intestinal function, exerting enduring effects on overall health (14–17). These microorganisms possess the capability to generate short-chain fatty acids by breaking down plant polysaccharides such as starch, cellulose, and hemicellulose (18, 19). These short-chain fatty acids contribute to thermogenesis and immune function (20, 21). Comprising bacteria, protozoa, fungi, and viruses, the balance of microbiota between commensal and pathogenic microorganisms is essential for maintaining animal health (22). Disruptions in the microbial community have been linked to the onset of various diseases (23–25).

The survival strategies of goats, as adaptable and widespread ruminants, are intricately linked to seasonal changes (26). Dynamic changes occur in bacterial communities in the rumen and the feces of ruminants as they experience different seasons (27). However, limited studies have investigated whether changes in microbiota influenced by ambient temperature are associated with immune responses. Since immunity increases concomitantly with changes in rumen and fecal microbiota, it may be assumed that the microbiota mediates the alterations in host immunity. Exploring the compositional differences between the bacterial communities of animals during the cold season (winter, cold stress) and warm season (spring, warm weather) may aid in identifying potential probiotics with immunomodulatory properties. Administering these potential probiotics to animals under stress could enhance their adaptation to cold stress, therefore, promoting production efficiency in extreme weather conditions. Nanjiang Yellow goats, a typical mountain-bred breed, exhibit robust adaptability to endure cold stress in Sichuan Province, China. In the current study, Nanjiang Yellow goats were selected as a model and sampled during the cold season and the warm season. We conducted analyses of serum immune factors, as well as the composition of rumen and fecal microbial communities, to explore the crosstalk between microbiota and innate immunity under cold stress.

## Materials and methods

### Animals and experiment design

This study was carried out following the regulations of Instructive Notions with Respect to Caring for Experimental Animals and following review and approval of the protocol by the Experimental Animal Management Committee of the Zhejiang University.

The goats were sourced from the Nanjiang Yellow Goat Original Breeding Farm located in Bazhong, China. They were housed in dedicated pens that were equipped with provisions for feeding with unrestricted access to drinking water, and all goats were in one block in the feeding room. Commercial concentrate was provided twice daily at 08:00 and 18:00 throughout the trial. The detailed nutrient composition of the commercial concentrate is available in the **Supplementary Table 1**. Ten healthy and well-conditioned Nanjiang Yellow goats (aged 6 months, similar body weight, male) were selected for sampling Blood, rumen, and fecal samples were collected from the goats in the cold season (at the beginning of January, average temperature: 0°C) and warm season (at end of March, average temperature:18°C). Goats with histories of disease were not included in the experiment.

### Sampling and analysis

The ground and walls of the building were treated with insect repellent. Disinfection was carried out using Bromo Germaine (CAS: 7281-04-1, China Pharmaceutical Group Co., Ltd., Beijing, China). The clinical condition of the animals was assessed and recorded. All animals were found to be in good health at both samplings with no signs of diarrhea.

The collection of rumen contents and fecal followed the methods described previously (28, 29). Approximately, 25 mL of rumen contents were collected from each goat. Rumen contents were collected from each goat using oral stomach tubes, 3 hours after morning feeding. The initial 5 mL of rumen contents from each sampling was discarded to eliminate potential saliva contamination. The remaining rumen contents were filtered through four layers of cheesecloth. Fecal samples were collected by rectal stimulation. Both rumen and fecal samples were rapidly frozen in liquid nitrogen and stored until DNA isolation and analysis. The collected samples were divided into four groups: rumen contents of the cold season (CR), rumen contents of the warm season (WR), fecal of the cold season (CF), and fecal of the warm season (WF).

3 mL of blood was collected from the jugular vein of each goat, 3 hours after morning feeding and before collecting rumen contents and fecal samples. The serum was separated from the collected blood by centrifugation at 1500 × g for 15 minutes. The separated serum was transferred to microcentrifuge tubes. The serum samples were stored at -80°C until analysis. Serum immunoglobulin A (IgA, H108-1-1), immunoglobulin G (IgG, H106-1-1), immunoglobulin M (IgM, H109-1-1), interleukin-2 (IL-2, H003-1-1), interleukin-4 (IL-4, H005-1-1), and interleukin-6 (IL-6, H007-1-1) were measured. Measurements were conducted at a wavelength of 450 nm using commercial ELISA kits (Nanjing Jiancheng Biotech, Jiangsu, China). The analysis followed the manufacturer’s protocol, with readings performed using a microtiter plate reader (BioTek, USA).

### 16S rRNA gene sequencing

Total genomic DNA from fecal samples and rumen contents was extracted. A commercial kit (Tiangen Biotech, Beijing, China) was used for DNA extraction. Bacteria were enzymatically lysed during the DNA extraction process. DNA concentration and purity were assessed using a 1% agarose gel. Amplification of DNA was performed using the primer set 341F/806R (341F: 5’-CCTATYGGGRBGCASCAG-3’, 806R: 5’-GGACTACNNGGGTATCTAAT-3’). The primers targeted the V3-V4 region of the bacterial 16S rRNA gene. Paired-ended sequencing (2×300 bp) was conducted on the Illumina MiSeq platform. The sequencing was carried out by standard procedures (Novogene Technology Co. Ltd., Tianjin, China) (28, 30).

### Processing of sequencing data

Following the methods previously described (31), raw sequencing reads from different samples were subjected to demultiplexing and quality filtering to obtain high-quality and valid data. Using QIIME2 (http://qiime.org) was utilized for bioinformatics analysis. Sequencing data from CR and WR groups were compared. The objective was to analyze differences in the rumen microbiota of goats under warm and cold-temperature conditions. Sequencing data from CF and WF groups were compared to analyze differences in the fecal microbiota among goats under warm and cold-temperature conditions. Microbiota analyses involve assessing microbial diversity, composition, and abundance in the rumen samples. Linear discriminant analysis Effect Size (LEfSe) was applied to determine differential abundance of bacterial taxa between different samples. The principal coordinate analysis (PCoA) and LEfSe analysis were performed using the Novomagic (https://magic.novogene.com).

### Statistical analysis

Paired t-tests were used for the statistical analyses of serum immune factors and microbial diversity. Data were presented as mean ± SEM. Spearman correlation analysis was conducted using SPSS software (version 19, SPSS Inc., Chicago, IL, USA) to explore the relationships between immune factors and bacterial taxa. Correlation heatmaps were generated using the R program and the pheatmap package. A significance level of *P* < 0.05 was considered statistically significant. The weighted gene co-expression network analysis (WGCNA) was carried out using the WGCNA package in R (version 4.0.2) to investigate the relationship between immune indices and microbiota profiling with the power at 4. The different colors were used to identify different modules. The relationship of the microbial composition of positive correlation modules in WGCNA results was further explored, and the network was drawn by Cytoscape (Version 3.8.0).

## Results

### Higher lgA and lower IL-2 and IL-6 in goats during the cold season

**Figure 1:**
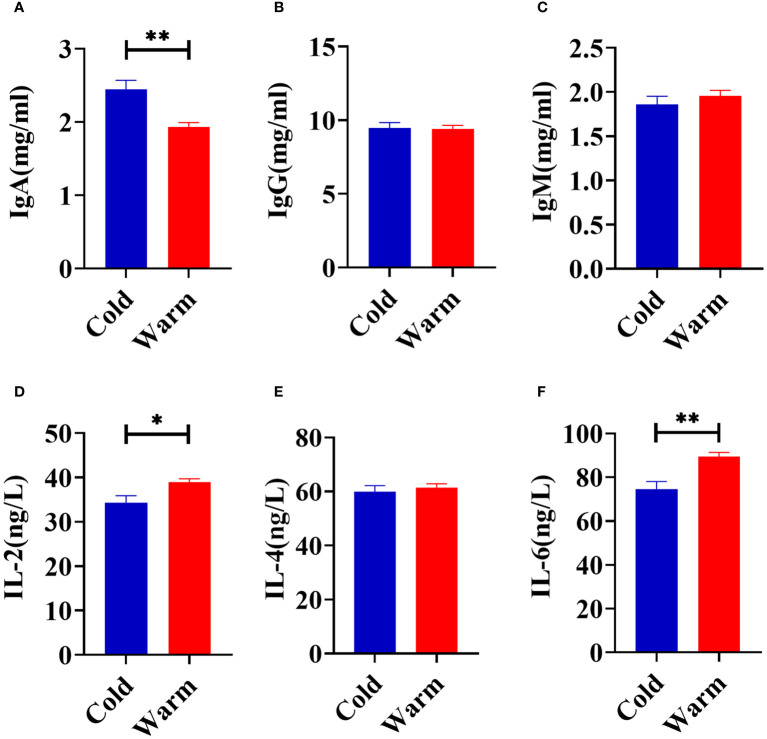
Comparison of serum immune factors concentrations of goats between cold and warm seasons. **(A)** The concentration of IgA. **(B)** The concentration of IgG. **(C)** The concentration of IgM. **(D)** The concentration of IL-2. **(E)** The concentration of IL-4. **(F)** The concentration of IL-6. The data were analyzed with paired t-tests, and the data were expressed as mean ± SEM. *P < 0.05 was statistically significance. **P < 0.01 was extremely significance.

Temperature changes significantly influenced the concentration of serum IgA (*P* < 0.01) with levels higher when goats were exposed to cold stress (**Figure 1A**). In comparison to the warm season, cold stress significantly reduced the serum concentrations of IL-2 (*P* = 0.02) and IL-6 (*P* < 0.01, **Figures 1D, F**). No significant differences in serum concentrations of IgG (*P* = 0.89), IgM (*P* = 0.42), and IL-4 (*P* = 0.56) were observed between the cold and warm seasons (**Figures 1B, C, E**).

### Comparison of rumen microbiota analysis between cold and warm seasons

**Figure 2:**
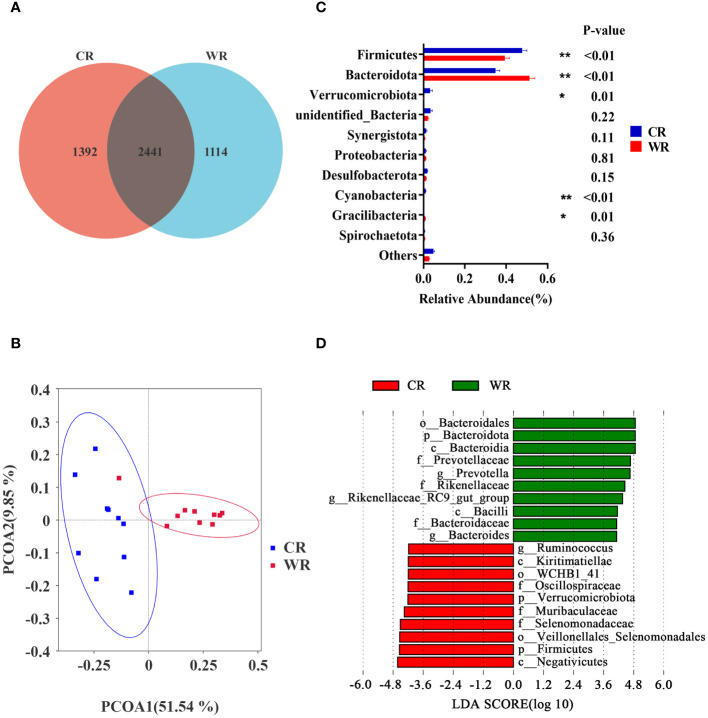
Comparison of rumen microbiota analysis between cold and warm seasons. **(A)** OTU Venn Diagram. **(B)** Principal coordinate analysis (PCoA) of microbial-based on weighted UniFrac. **(C)** Relative abundance of top10 phyla, *P<0.05, **P<0.01. **(D)** The bar chart shows the LDA scores of goats. LDA score > 4. CR: rumen of goats of the cold season. WR: rumen of goats of the warm season.

Amplification sequencing of the 16S rRNA gene in rumen samples identified 5103 operational taxonomic units (OTUs, **Supplementary Table 2**). After removing OTUs annotated as Archaea and unannotated OTUs, there were 5081 OTUs annotated as Bacteria. The rarefaction curves (**Supplementary Figure 1A**) gradually flattened out, indicating an even distribution of species and a reasonable amount of sequencing data for subsequent analysis. The Venn analysis showed that the CR and WR groups shared 2441 OTUs, and 1392 and 1114 OTUs were uniquely detected, respectively (**Figure 2A**). PCoA of OTUs showed a clear clustering of samples by temperature variation (PCoA1 = 51.54%, PCoA2 = 9.85%, **Figure 2B**, **Supplementary Figure 1B**). However, there were no significant differences in Chao1, Simpson, and Shannon indices between the two groups, indicating that temperature changes did not significantly affect microbial species diversity in the rumen (**Supplementary Figure 1C**).

Based on the data at the phylum level, the dominant phyla in the CR and WR groups were *Firmicutes* and *Bacteroidota*. As shown in **Figure 2C**, the CR group exhibited higher levels of *Firmicutes* (*P*< 0.01), *Verrucomicrobiota* (*P* = 0.01), and *Cyanobacteria* (*P* < 0.01) compared with the WR group. In contrast, the CR group had lower levels of *Bacteroidota* (*P* < 0.01) and *Gracilibacteria* (*P* = 0.01) compared with the WR group. To determine the differences in abundance of bacterial taxa between CR and WR, LEfSe were employed with Linear Discriminant Analysis (LDA) score > 4 (**Figure 2D**). The WR group displayed a higher abundance of g_*Prevotella*, g_*Rikenellaceae*_RC9_gut_group, and g_*Bacteroides*, while the CR group exhibited a higher abundance of g*_Ruminococcus*.

### Comparison of fecal microbiota analysis between cold and warm seasons

**Figure 3:**
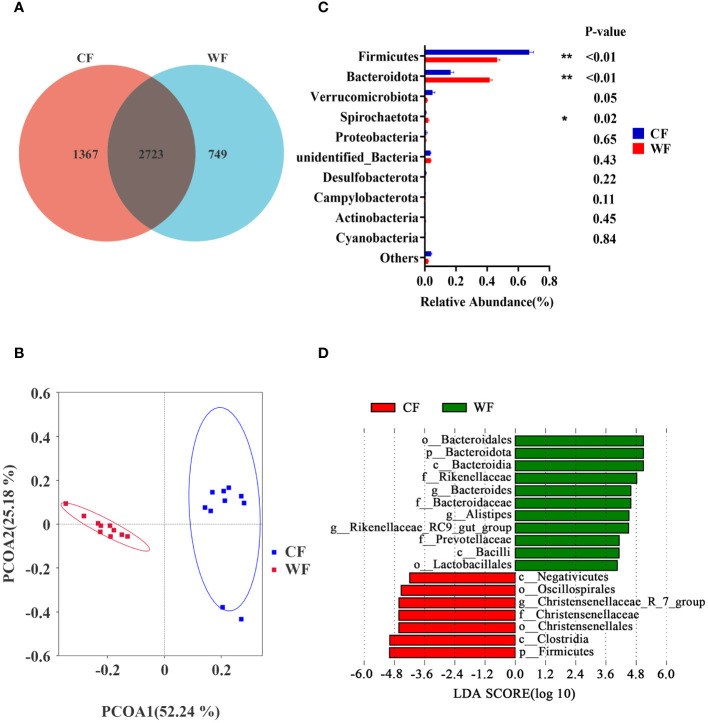
Comparison of fecal microbiota analysis between cold and warm seasons. **(A)** OTU Venn Diagram. **(B)** Principal coordinate analysis (PCoA) of microbial-based on weighted UniFrac. **(C)** Relative abundance of top10 phyla, *P<0.05, **P<0.01. **(D)** The bar chart shows the LDA scores of goats. LDA score > 4. CF: fecal of goats of the cold season. WF: fecal of goats of the warm season.

Amplification sequencing of the 16S rRNA gene in fecal samples identified 5005 operational taxonomic units (OTUs, **Supplementary Table 3**). After removing OTUs annotated as Archaea and unannotated OTUs, there were 4980 operational taxonomic units annotated as Bacteria. The rarefaction curves gradually leveled off (**Supplementary Figure 1D**). The Venn analysis showed that the CF and WF groups shared 2723 OTUs, and 1367 and 749 OTUs were uniquely detected, respectively (**Figure 3A**). PCoA analysis of OTUs showed a clear clustering of samples by temperature variation (PCoA1 = 52.24%, PCoA2 = 25.18%, **Figure 3B**, and **Supplementary Figure 1E**). However, there was no significant difference in the Chao1, Simpson, and Shannon indices between the two groups (**Supplementary Figure 1F**).

The *Firmicutes* and *Bacteroidota* are the main phyla shared by CF and WF groups. As shown in **Figure 3C**, the CF group had a higher abundance of *Firmicutes* (*P* < 0.01) compared to the WF group. Additionally, the CF group had lower levels of *Bacteroidota* (*P* < 0.01) and *Spirochaetota* (*P* = 0.02) compared with the MF group. The LEfSe analysis, with LDA score > 4, showed that g_*Bacteroides*, g_*Alistipes*, and g_*Rikenellaceae*_RC9_gut_group were more abundant in the WF, while g_ *Christensenellaceae*_R_7_group was more abundant in the CF group (**Figure 3D**).

### Alterations of rumen-fecal microbiota under cold stress are associated with concentrations of immune factors

**Figure 4:**
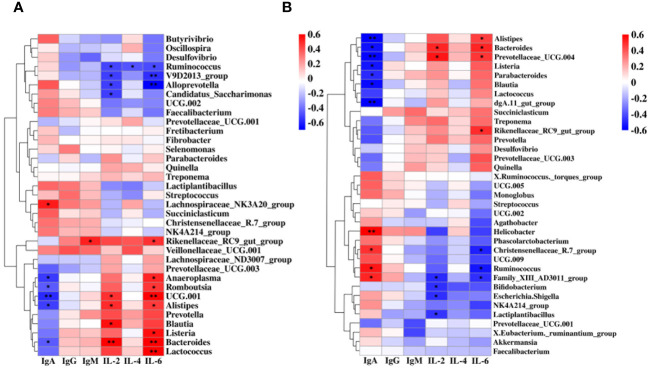
Correlation between the immune factors and the rumen and fecal bacteria at the genus level. **(A)** Spearman correlation between rumen samples (CR, WR) and serum immune factors. **(B)** Spearman correlation between fecal samples (CF, WF) and serum immune factors. *P < 0.05, **P < 0.01.

To determine the association between feces-rumen bacteria and concentration of IgA, IgG, IgM, IL-2, IL-4, and IL-6, spearman correlation analyses were conducted at the genera level. In the rumen, correlation analysis revealed a positive correlation between IgA and *Lachnospiraceae*_NK3A20_group (*P* = 0.02, R = 0.53). It was also observed that IL-2 showed a positive correlation with four bacterial genera, including UCG.001 (*P* = 0.04, R = 0.47), *Alistipes* (*P* = 0.03, R = 0.49), *Blautia* (*P* = 0.01, R = 0.55), and *Bacteroides* (*P* < 0.01, R = 0.60). It was found that eight bacterial genera were positively associated with IL-6, including *Rikenellaceae*_RC9_gut_group (*P* = 0.03, R = 0.48), *Anaeroplasma* (*P* = 0.02, R = 0.50), *Romboutsia* (*P* = 0.04, R = 0.47), UCG.001 (*P* < 0.01, R = 0.58), *Alistipes* (*P* = 0.05, R = 0.45), *Listeria* (*P* = 0.01, R = 0.55), *Bacteroides* (*P* < 0.01, R = 0.59), and *Lactococcus* (*P* < 0.01, R = 0.63). Five genera in the rumen showed a negative correlation with serum IgA, including *Anaeroplasma* (*P* = 0.02, R = -0.53), *Romboutsia* (*P* = 0.04, R = -0.46), UCG.001 (*P* < 0.01, R = -0.58), *Alistipes* (*P* = 0.02, R = -0.52), and *Bacteroides* (*P* = 0.05, R = -0.45). Four genera in the rumen were negatively correlated with IL-2, including *Ruminococcus* (*P* = 0.02, R = -0.51), V9D2013_group (*P* = 0.02, R = -0.53), *Alloprevotella* (*P* = 0.02, R = -0.52), and *Candidatus_Saccharimonas* (*P* = 0.02, R = -0.52). *Ruminococcus* (*P* = 0.03, R = -0.49), V9D2013_group (*P* < 0.01, R = -0.57), and *Alloprevotella* (*P* < 0.01, R = -0.71) were negatively correlated with IL-6 (**Figure 4A**).

Four genera were also positively associated with IgA in feces, including *Helicobacter* (*P* < 0.01, R = 0.63), *Christensenellaceae*_R.7_group (*P* = 0.03, R = 0.49), *Ruminococcus* (*P* = 0.01, R = 0.54), and *Family*_XIII_AD3011_group (*P* = 0.02, R = 0.51). *Bacteroides* (*P* = 0.02, R = 0.51) and *Prevotellaceae*_UCG.004 (*P* = 0.01, R = 0.54) were positively correlated with IL-2. While *Alistipes* (*P* = 0.05, R = 0.45), *Bacteroides* (*P* = 0.02, R = 0.51), *Prevotellaceae*_UCG.004 (*P* = 0.03, R = 0.48), and *Rikenellaceae*_RC9_gut_group (*P* = 0.03, R = 0.50) were positively correlated with IL-6. In addition, seven genera were negatively associated with IgA in feces, including *Alistipes* (*P* < 0.01, R = -0.62), *Bacteroides* (*P* = 0.01, R = -0.54), *Prevotellaceae*_UCG.004 (*P* < 0.01, R = -0.57), *Listeria* (*P* = 0.02, R = -0.53), *Parabacteroides* (*P* = 0.03, R = -0.49), *Blautia* (*P* = 0.01, R = -0.55), and *dgA*.11_gut_group (*P* < 0.01, R = -0.58). Four genera were negatively correlated with IL-2, including *Family*_XIII_ AD3011_group (*P* = 0.03, R = -0.48), *Bifidobacterium* (*P* = 0.04, R = -0.47), *Escherichia.Shigella* (*P* = 0.03, R = -0.48), and *Lactiplantibacillus* (*P* = 0.04, R = -0.45). *Christensenellaceae*_R.7_group (*P* = 0.02, R = -0.50), *Ruminococcus* (*P* = 0.01, R = -0.54), and *Family*_XIII_AD3011_group (*P* = 0.03, R = -0.48) were negatively correlated with IL-6 (**Figure 4B**).

### Core microbiota is associated with the alterations of immune factors under cold stress

**Figure 5:**
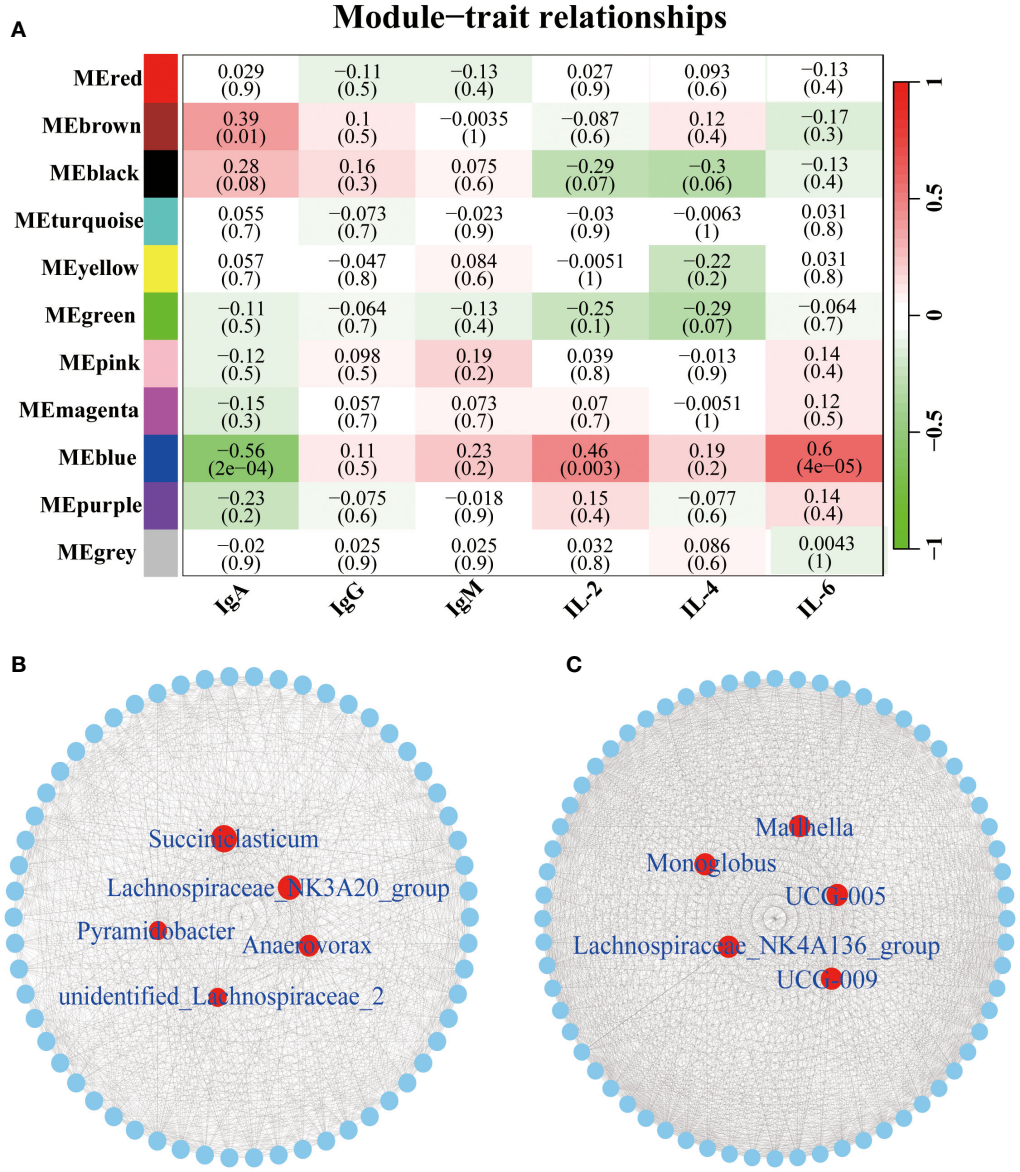
Correlation between the serum immune factors and abundance of the microbiome in rumen and fecal at the genus level. The correlated module was analyzed using a weighted gene co-expression network analysis (WGCNA). Correlation networks were generated using Spearman’s rank correlation coefficients and visualized using the Cytoscape. **(A)** The heatmap of the WGCNA module. **(B)** Microbiome interaction network and core microbes in the MEbrown module. Microbiota that make up the network are listed in the **Supplementary Table 4**. **(C)** Microbiome interaction network and core microbes in the MEblue module. Microbiota that make up the network are listed in the **Supplementary Table 5**.

To identify the core microbiota altering the concentrations of immune factors, we further performed the WGCNA analysis. A total of 11 relevant microbiota modules were identified (**Figure 5A**). Among them, the MEbrown module was significantly associated with IgA (*P* = 0.01, R = 0.39). While MEBlue module was significantly correlated with IgA (*P* < 0.01, R = -0.56), IL-2 (*P* < 0.01, R = 0.46), and IL-6 (*P* < 0.01, R = 0.60). The network exported from the MEbrown module showed that *Succiniclasticum*, *Lachnospiraceae*_NK3A20_group, *Anaerovorax*, *Pyramidobacter*, and *unidentified*_*Lachnospiraceae*_2 was the core microbiota in the MEbrown module (**Figure 5B**). Networks derived from the MEblue module showed that UCG-005, *Monoglobus*, UCG-009, *Lachnospiraceae*_NK4A136_group, and *Mailhella* were the central microbiota in the MEblue module (**Figure 5C**).

## Discussion

The stress resistance of organisms is of great significance to their survival. Young animals, characterized by their immature immune systems, often display heightened susceptibility to challenges posed by cold stress. This susceptibility can lead to microbiome imbalances, making young animals an ideal model to study the crosstalk between microbial profiles and host immune responses (32, 33). In the current study, we measured the profiles of rumen and feces microbiota to investigate their relationship with innate immunity in goats. Notable shifts in microbial distribution and concentrations of IgA were observed during the transition from cold to warm seasons. These findings underscore a crosstalk between microbiota compositions and innate immunity under cold stress in goats.

In order to maintain overall health, the immune system of ruminants actively combats pathogenic microbiota by secreting immunoglobulins (34, 35). The concentrations of these immunoglobulins in serum serve as direct indicators of resistance to external pathogenic microbiota (36). IgA is crucial for mucosal immunity and helps protect the body from infections (37). In the current study, the elevated concentrations of IgA in goats under cold stress suggest an active immune response. IL-2 is essential for the activation and growth of T cells, while IL-6 is involved in regulating the immune response (38, 39). The lower levels of IL-2 and IL-6 observed during the cold season suggest a compromised immunity. These observations are consistent with the increased susceptibility of goats to diseases during colder seasons (40). Understanding these immune responses is crucial for implementing strategies to support the health and well-being of ruminants, particularly during challenging environmental conditions.

The study revealed that the diversity of the rumen microbial community showed no significant difference between the cold and warm seasons, which could be attributed to the ongoing maturation of the rumen in young goats. Despite this, there were notable alterations in the compositions of the rumen microbiota. Consistent with previous findings, the dominant phyla in the rumen microbial community during both seasons were *Firmicutes* and *Bacteroidota* (29). Interestingly, there was a heightened abundance of *Verrucomicrobiota* in the cold season, aligning with its role in polysaccharide degradation, crucial for meeting the energy requirements of the host (41, 42). The study also observed variations in the abundance of disease-protection-associated genera, such as *Prevotella* (43) and *Bacteroides* (44), with higher prevalence in the rumen during the warm season, implying heightened immunity. This was confirmed by positive correlations between the abundance of four genera in the rumen and IL-2 concentrations, as well as the positive correlations between the abundance of eight genera and IL-6 concentrations. Among these genera, *Alistipes* (45), *Bacteroides* (44), and *Blautia* (46), at least, have associations with inflammation and disease protection. This suggests that the modulation of these microbiota could potentially enhance host immunity under cold stress.

The diversity of gut microbes has emerged as a novel marker for evaluating gut health and metabolic capacity (47). Interestingly, we found no significant differences in gut microbiome diversity between the cold and warm seasons, suggesting a certain resilience of fecal microbes to variations in ambient temperature. Similar to the data of rumen, the dominant phyla in the feces during both seasons were *Firmicutes* and *Bacteroidota*, known for their pivotal roles in carbohydrate and protein metabolism (48). Notably, there was a lower abundance of *Alistipes* and *Bacteroides* in the feces under cold stress, implying a diminishing immune response at this stage. *Ruminococcus*, positive correlation with IgA concentrations, plays a role in the degradation of complex polysaccharides, converting them into nutrients for the host (49). Among the six genera positively correlated with IL-2 and IL-6 concentrations, *Alistipes*, *Bacteroides*, and *Prevotellaceae*_UCG.004 were associated with increasing antioxidant performance in sheep (50). These findings highlight a crosstalk between rumen and feces microbiota and the immune response in goats under cold stress.

The development of additives with immunomodulatory properties is proposed as a valuable strategy to enhance the production efficiency of animals under cold stress (51). positive correlation between the MEbrown module and IgA is supported by the dominance of *Succiniclasticum* (52), *Lachnospiraceae_*NK3A20_group (53), and *Pyramidobacter* (54), which are associated with nutrient metabolism. This aligns with the concept of increased energy metabolism and nutrient demands in goats during cold stress. Additionally, the MEblue module, particularly the presence of *Lachnospiraceae*_NK4A136_group, is linked to the production of short-chain fatty acids, known to improve the intestinal epithelial barrier and inhibit inflammation (55). This group exhibits a positive correlation with IL-2 and IL-6. Collectively, these findings suggest that microbiota associated with immunity plays a pivotal role in maintaining the health of goats under cold stress. However, it is emphasized that further studies are needed to comprehensively evaluate the immune-related bacteria identified in this study. The potential development of additives with immunomodulatory properties could prove beneficial to fortify the immunity of animals and enhance their ability to withstand the challenges posed by cold stress.

## Conclusion

The study comprehensively investigated the correlation between rumen-fecal microbiota and the immune response in goats under cold stress. Notably, we observed a decrease in IL-2 and IL-6 and an increase in IgA during cold stress. While acknowledging that measuring specific antibodies against bacteria would provide a more nuanced understanding of the host immune response, our data identified eight genera in the rumen and four genera in the feces that positively correlated with changes in immune factors. The positive correlations between certain probiotics, including *Alistipes*, *Bacteroides*, *Blautia*, and *Prevotellaceae*_UCG.004, and IL-2, and IL-6 suggest their potential role in immunomodulatory properties. However, further experiments are necessary to elucidate the mechanisms that enhance tolerance to cold stress. Collectively, our study underscores the crosstalk between rumen-fecal microbiota and innate immune responses under cold stress in goats. Identifying these microbiotas with immunomodulatory properties is crucial for developing strategies to enhance the production efficiency of animals facing cold stress.

## Data availability statement

The datasets presented in this study can be found in online repositories. The names of the repository/repositories and accession number(s) can be found below: https://www.ncbi.nlm.nih.gov/, PRJNA1050866.

## Ethics statement

The animal study was approved by The Experimental Animal Management Committee of the Zhejiang University. The study was conducted in accordance with the local legislation and institutional requirements.

## Author contributions

TL: Data curation, Project administration, Software, Writing – original draft. JZ: Project administration, Writing – review & editing. KL: Data curation, Software, Writing – review & editing. YL: Project administration, Software, Writing – review & editing. JL: Project administration, Software, Writing – review & editing. YC: Project administration, Software, Writing – review & editing. HS: Funding acquisition, Writing – review & editing, Conceptualization, Project administration, Supervision.

## Supplementary material

Supplementary Table Nutrient compositions of the commercial concentrate for goats.

Supplementary File 1 OTUs of rumen samples.

Supplementary File 2 OTUs of fecal samples.

Supplementary File 3 The remaining microbiota that makes up the network of the MEbrown module.

Supplementary File 4 The remaining microbiota that makes up the network of the MEblue module.

Supplementary Figure 1 Rarefaction curves **(A)**, PCoA based on unweighted UniFrac distances **(B)**, and alpha index (Chao1, Simpson, and Shannon) **(C)** of CR and WR. Rarefaction curves **(D)**, PCoA based on unweighted UniFrac distances **(E)**, and alpha index (Chao1, Simpson, and Shannon) **(F)** of CF and WF. CR: rumen of goats of the cold season. WR: rumen of goats of the warm season. CF: fecal of goats of the cold season. WF: fecal of goats of the warm season.
