# Supplementary material for: Crosstalk between innate immunity and rumen-fecal microbiota under the cold stress in goats
Source: Front Immunol. 2024 Feb 26;15:1363664. doi: 10.3389/fimmu.2024.1363664 (PMC10928366; doi:10.3389/fimmu.2024.1363664)
Supplement: Supplementary Table — Nutrient compositions of the commercial concentrate for goats. [file Table_1.docx]

**Table S1. Nutrient compositions of the commercial concentrate.**

| *Nutrient composition ( %)* | |
| --- | --- |
| Crude protein (%) | ≥15 |
| Crude fibre (%) | ≤15 |
| Crude ash (%) | ≤10 |
| Ca (%) | 0.5-2.0 |
| P (%) | 0.4 |
| NaCl (%) | 0.3-1.5 |
| H_2_O (%) | ≤14 |
