# Supplementary figures and images for: Crosstalk between innate immunity and rumen-fecal microbiota under the cold stress in goats
Source: Front Immunol. 2024 Feb 26;15:1363664. doi: 10.3389/fimmu.2024.1363664 (PMC10928366; doi:10.3389/fimmu.2024.1363664)

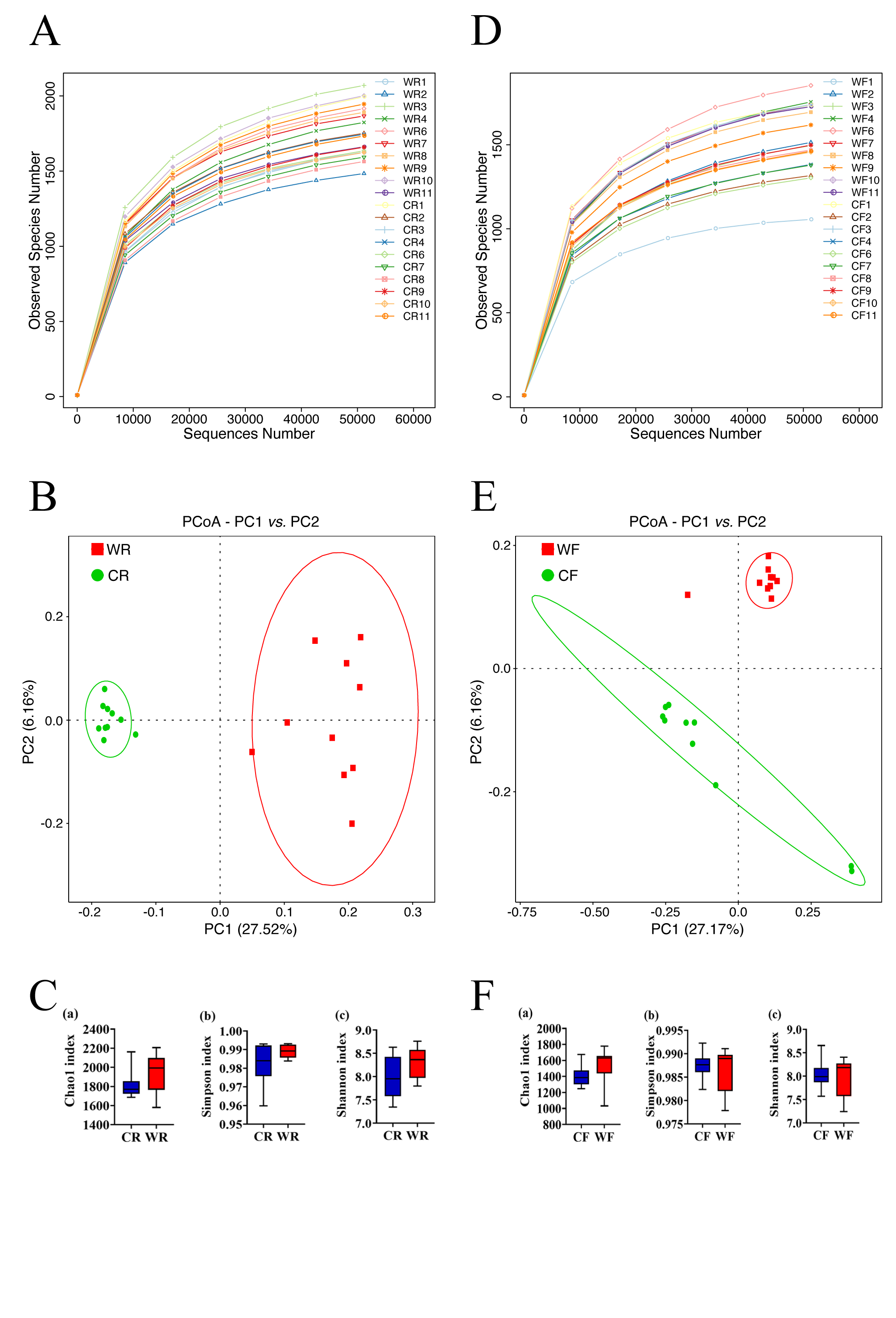

Supplement: Supplementary Figure 1 — Rarefaction curves (A), PCoA based on unweighted UniFrac distances (B), and alpha index (Chao1, Simpson, and Shannon) (C) of CR and WR. Rarefaction curves (D), PCoA based on unweighted UniFrac distances (E), and alpha index (Chao1, Simpson, and Shannon) (F) of CF and WF. CR: rumen of goats of the cold season. WR: rumen of goats of the warm season. CF: fecal of goats of the cold season. WF: fecal of goats of the warm season. [file Image_1.tif]
